# A student’s perspective on the effectiveness of strategies to overcome barriers of critical thinking in medical students’ curriculum 

**DOI:** 10.30476/jamp.2020.86346.1232

**Published:** 2021-07

**Authors:** KHULAT SAQI, SAMINEH YOUSEFI, ZOYA MEHDI, LAILA HAZARA

**Affiliations:** 1 Medical School, Faculty of Life Sciences and Medicine, King's College London, London, UK

## Dear Editor

We appreciate the research by Kasalaei et al. ( 1 ) in analysing the barriers to critical thinking in medical school curriculums. As current medical students at King’s College in London, we would like to discuss how our medical school has addressed some of the barriers mentioned and has consequently enhanced our critical thinking skills.

Ineffective evaluation within the learning environment is suggested by the authors as one such barrier. Without receiving appropriate feedback and discussing potential improvements in academic work, students cannot enhance their learning experience or engage in higher level analytical reflection ( 1 ). In particular, one form of evaluation, peer feedback, has been widely adopted by our medical school. As current students, we believe that this has been very beneficial in allowing us to gain an insight into the different ways our peers have approached the same task, and literature has also shown it has positive effects on performance ( 2 ). Furthermore, from personal experience, the active process of engaging in peer feedback is highly useful in familiarizing the students with the marking criteria and steps involved in high achievements in the set task. This idea has been supported by the literature ( 3 ).

Due to resource constraints, traditional teaching methods are often prioritized as they are viewed as less time-consuming, which has created a difficult environment for critical thinking skills to be taught ( 1 ). The innovative practice of flipped classrooms adopted by King’s College London, allows for a collaborative learning environment where students are encouraged to participate in discussion and develop skills of critical analysis. Students have the opportunity to learn independently outside the classroom via resources such as lecture notes or supplementary online videos. This enables more time in class for these discussions, improving teacher student interaction. Creating a learner-centred environment such as this promotes an active form of learning which has been shown to increase learning motivation and engagement of students ( 4 , 5 ).

Furthermore, our medical school has incorporated a peer teaching module into the curriculum, designed to equip its students with the necessary skillset to be effective educationalists. We believe we have greatly benefitted both from being taught by and by teaching our peers. Through the process of creating lesson plans and experimenting with different learning techniques, we have been able to improve such qualities as leadership and confidence. Kasalaei et al. ( 1 ) suggest that lack of confidence in itself can be a barrier to the development of critical thinking skills, and this may be a step towards mitigating this. The literature also suggests that peer teaching helps to create a comfortable and supportive environment which allows for optimal learning and relieve pressure from the faculty staff ( 6 ).

Ultimately, finding a way to encourage innovation within the education sector and break the mould of purely traditional teaching patterns appears to be the way forward. The examples discussed in this letter give an insight into some of the strategies which we believe should be more widely adopted by medical schools.
